# Liu Jun Zi Tang—A Potential, Multi-Herbal Complementary Therapy for Chemotherapy-Induced Neurotoxicity

**DOI:** 10.3390/ijms19041258

**Published:** 2018-04-23

**Authors:** Chun-Tang Chiou, Kaw-Chen Wang, Ying-Chen Yang, Chuen-Lin Huang, Sien-Hung Yang, Yao-Haur Kuo, Nai-Kuei Huang

**Affiliations:** 1 National Research Institute of Chinese Medicine, Ministry of Health and Welfare, Taipei 11031, Taiwan; ctchiou@nricm.edu.tw (C.-T.C.); kuoyh@nricm.edu.tw (Y.-H.K.); 2 Department of Neurology, Cardinal-Tien Hospital, New Taipei City 231, Taiwan; aronkcwang@gmail.com; 3 Department of Biotechnology and Animal Science, National Ilan University, Ilan 260, Taiwan; ycyang@niu.edu.tw; 4 Medical Research Center, Cardinal Tien Hospital, Hsintien, New Taipei City 231, Taiwan; chuenlinhuang@hotmail.com; 5 Graduate Institute of Physiology & Department of Physiology and Biophysics, National Defense Medical Center, Taipei 11031, Taiwan; 6 School of Traditional Chinese Medicine, Chang Gung University, Taoyuan City 333, Taiwan; dryang@adm.cgmh.org.tw; 7 Graduate Institute of Integrated Medicine, College of Chinese Medicine, China Medical University, Taichung 404, Taiwan; 8 Ph.D. Program for Neural Regenerative Medicine, College of Medical Science and Technology, Taipei Medical University, Taipei 11031, Taiwan

**Keywords:** Liu Jun Zi Tang, cisplatin, neurotoxicity, mitochondria, PGC-1α

## Abstract

Liu Jun Zi Tang (LJZT) has been used to treat functional dyspepsia and depression, suggesting its effects on gastrointestinal and neurological functions. LJZT is currently used as a complementary therapy to attenuate cisplatin-induced side effects, such as dyspepsia. However, its effect on chemotherapy-induced neuropathic pain or neurotoxicity has rarely been studied. Thus, we explored potential mechanisms underlying LJZT protection against cisplatin-induced neurotoxicity. We observed that LJZT attenuated cisplatin-induced thermal hyperalgesia in mice and apoptosis in human neuroblastoma SH-SY5Y cells. Furthermore, it also attenuated cisplatin-induced cytosolic and mitochondrial free radical formation, reversed the cisplatin-induced decrease in mitochondrial membrane potential, and increased the release of mitochondrial pro-apoptotic factors. LJZT not only activated the peroxisome proliferator-activated receptor gamma coactivator 1-alpha (*PGC-1α*) promoter region, but also attenuated the cisplatin-induced reduction of *PGC-1α* expression. Silencing of the *PGC-1α* gene counteracted the protection of LJZT. Taken together, LJZT mediated, through anti-oxidative effect and mitochondrial function regulation, to prevent cisplatin-induced neurotoxicity.

## 1. Introduction

Liu Jun Zi Tang (LJZT), a famous Chinese herbal formula and Kampo medicine, known as Rikkunshito in Japan, has been used for several hundred years. LJZT was first described in the medical book, *Fu Ren Da Quan Liang Fang* (*Compendium of Excellent Treatments for Women*), compiled by the herbalist Chen Zi Ming. Its indications for use include spleen-stomach qi deficiency-induced phlegm and dyspepsia, and chest and epigastric oppression and distress [1]. Modern research has evidenced the effects of LJZT in regulating gastrointestinal functions [2]. Thus, LJZT and its related decoctions have recently been intensively investigated as complementary therapies for chemotherapy-induced side effects [3,4,5,6], such as cisplatin-induced dyspepsia and cancer anorexia-cachexia syndrome [7]. Furthermore, LJZT and its related decoctions have also been demonstrated to elicit neurological and pain-relieving functions, such as alleviating depression [8] and suppressing abdominal pain [2].

Chemotherapy-induced peripheral neuropathy is the most common neurological complication associated with cancer treatment, with vincristine, paclitaxel, oxaliplatin, and cisplatin being the most neurotoxic compounds [9]. This neurotoxicity may lead to disability and decreased quality of life. The most common symptoms involve pain, burning, tingling, loss of sensation, and balance problems. Further, the painful sensations may involve shooting or electric shock-like pain, non-evoked burning, as well as mechanical or thermal allodynia or hyperalgesia [10]. Among these medications, cisplatin has played a major role in cancer chemotherapy for many years and is a commonly-used antineoplastic drug in clinical treatment [11]. Cisplatin exerts its antitumor activity by binding with DNA and distorting its helical structure, resulting in transcription inhibition [12] and apoptosis through the pathways of DNA damage recognition [13].

Although cisplatin induces ototoxicity, myelotoxicity, renal toxicity, and gastrointestinal toxicity [14,15] through apoptotic events, the mechanism underlying cisplatin-induced peripheral neurotoxicity is still unclear. Apart from adduction to nuclear DNA, its binding to mitochondrial DNA and proteins is generally regarded as the key event in initiating apoptosis [16]. Mitochondrial dysfunction is a crucial function of cisplatin in initiating apoptosis in the dorsal root ganglia [17], which contained the highest concentration of cisplatin in a postmortem study [18], and was thus proposed as a possible mechanism underlying neuropathy [19]. Therefore, in recent years, the role of mitochondrial function in mediating neurotoxicity has been intensively investigated [20,21].

Although LJZT is known to alleviate chemotherapy-induced gastrointestinal side effects, its neurological and pain-relieving functions have rarely been investigated. Thus, in this study, potential mechanisms underlying LJZT protection against cisplatin-induced neurotoxicity were explored.

## 2. Results

### 2.1. LJZT Prevented Cisplatin-Induced Thermal Hyperalgesia in Mice and Apoptosis in Human Neuroblastoma SH-SY5Y Cells

**Figure 1:**
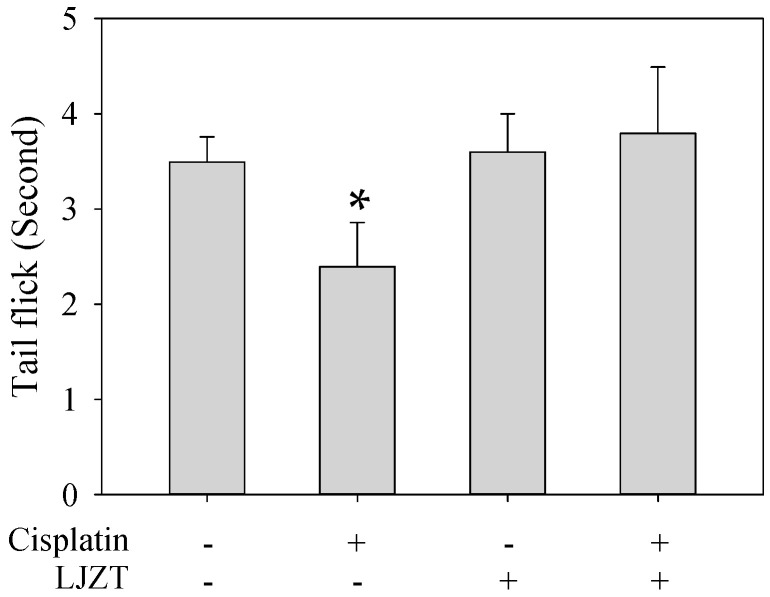
Liu Jun Zi Tang attenuated cisplatin-induced thermal hyperalgesia in mice. Eight-week-old Balb/c mice were housed in a cage and habituated for two weeks to avoid stress-induced analgesia. Cisplatin was freshly prepared in normal saline. Mice were treated with daily intraperitoneal injections for 5 days, followed by 9 days of rest, for two cycles. Liu Jun Zi Tang (LJZT) was administered 1 h prior to cisplatin administration. After the treatments, a tail-flick assay was performed to monitor tolerance time in response to heat. Data points represented the mean tolerance time ± standard deviation. Differences among multiple groups were evaluated using one-way analysis of variance. Differences between means were calculated using the Student-Newman-Keuls method and were considered significant at *p* < 0.05. * *p* < 0.05, compared with controls (*n* = 6–8).

**Figure 2:**
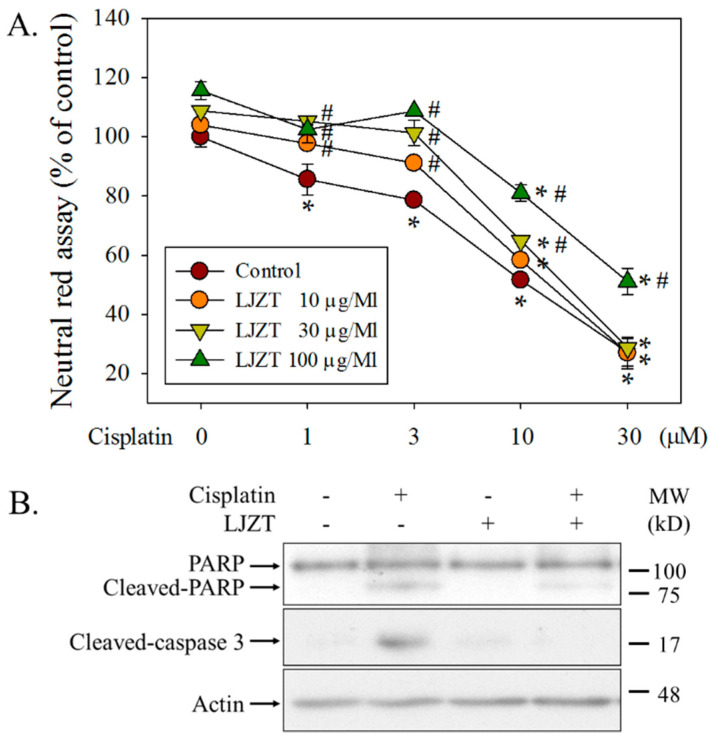
Effects of Liu Jun Zi Tang on cisplatin-induced cell cytotoxicity in human neuroblastoma (SH-SY5Y) cells. (**A**) Different doses of cisplatin were administered for another 24 h with or without pretreatment of different doses of LJZT for 1 h. Cells were then subjected to a neutral red assay. Viability was expressed as a percentage of controls, and data are presented as the mean ± standard deviation. Differences among multiple groups were evaluated through two-way ANOVA. Differences between means were calculated using the Student-Newman-Keuls method with significance set at *p* < 0.05. * *p* < 0.05, compared with controls (*n* = 3–6). # *p* < 0.05, compared with the cisplatin-only treatment group (*n* = 3–6). (**B**) After pretreatment with or without LJZT, cells were treated with or without cisplatin for another 24 h. Cells were then harvested and subjected to a Western blot analysis.

LJZT significantly reversed cisplatin-induced thermal hyperalgesia in a mouse model of neuropathy (Figure 1). Cisplatin dose-dependently caused significant cell death in human SH-SY5Y cells (Figure 2A) and differentially protected against cisplatin-induced cell death. Additionally, LJZT was demonstrated to attenuate cisplatin-induced cleavages of poly (ADP-ribose) polymerase (PARP) and caspase 3 (Figure 2B). Because treatment with 10 µM of cisplatin resulted in approximately 50% cell death and 100 µg/mL of LJZT exerted the highest protective effect, these two dosages were adopted in this study.

### 2.2. LJZT Prevented Cisplatin-Induced Cytosolic Free Radical Formation

**Figure 3:**
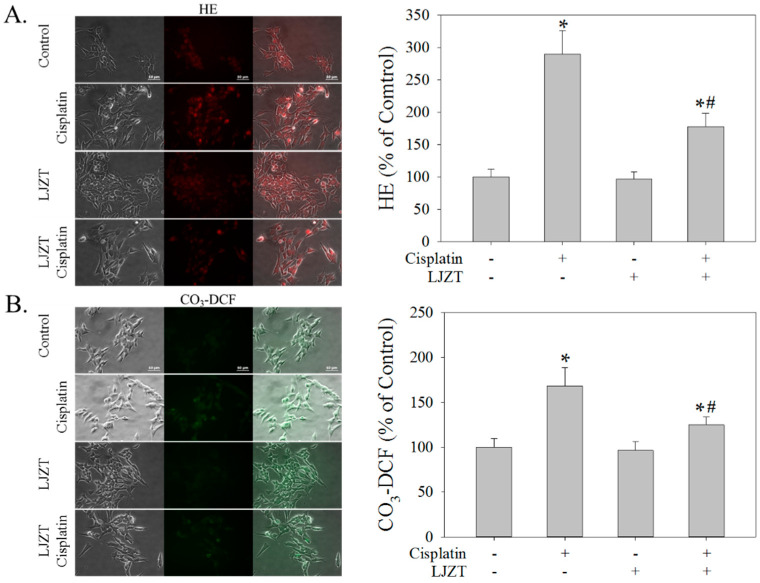
Effects of Liu Jun Zi Tang on cisplatin-induced cytosolic reactive oxygen species formation in human neuroblastoma (SH-SY5Y) cells. Cisplatin was administered for another 24 h with or without pretreatment with LJZT for 1 h. Cells were then stained with (**A**) di-hydroxyethidium (DHE) and (**B**) CO_3_-H_2_DCFDA and subjected to image acquisition and quantitation. At least 10 frames were randomly acquired for each group. Fluorescence intensity is expressed as a percentage of controls. Data are presented as the mean ± standard deviation. Differences among multiple groups were evaluated using one-way analysis of variance and those between means were calculated using the Student-Newman-Keuls method with significance set at *p* < 0.05. * *p* < 0.05, compared with controls (*n* = 3–6). # *p* < 0.05, compared with the cisplatin-only treatment group (*n* = 3–6). The bar represents 50 µm. These data represent one out of three independent experiments that provided similar results.

LJZT significantly attenuated the cisplatin-induced increase in the fluorescent intensities of 2-hydroxyethidium (HE, Figure 3A) and CO_3_-DCF (Figure 3B).

### 2.3. LJZT Attenuated Cisplatin-Induced Mitochondrial Dysfunction

**Figure 4:**
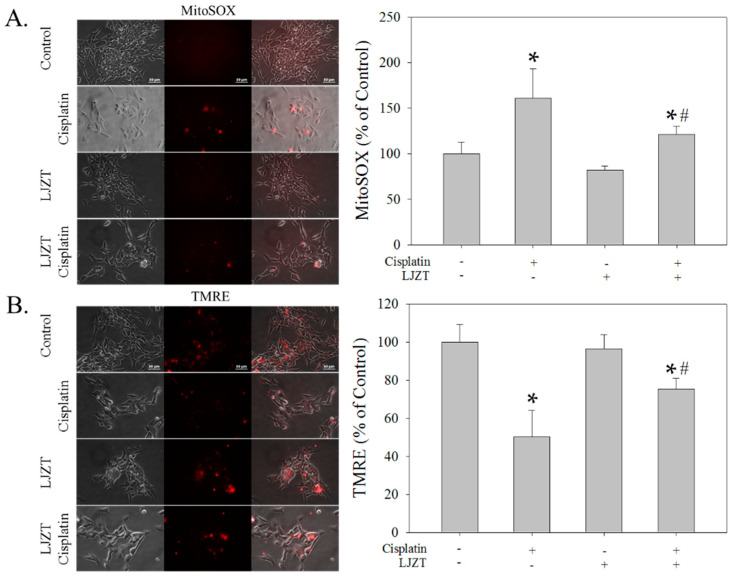
Effects of Liu Jun Zi Tang on cisplatin-induced mitochondrial reactive oxygen species formation and membrane potential in human neuroblastoma (SH-SY5Y) cells. Cisplatin was administered for another 24 h with or without pretreatment with LJZT for 1 h. Cells were then stained with (**A**) MitoSOX and (**B**) tetramethylrhodamine, ethyl ester (TMRE), and subjected to image acquisition and quantitation. At least 10 frames were randomly acquired for each group. Fluorescence intensity is expressed as a percentage of controls. Data are presented as the mean ± standard deviation. Differences among multiple groups were evaluated using one-way analysis of variance, and those between means were calculated using the Student-Newman-Keuls method with significance set at *p* < 0.05. * *p* < 0.05, compared with controls (*n* = 3–6). # *p* < 0.05, compared with the cisplatin-only treatment group (*n* = 3–6). The bar represents 50 µm. These data represent one out of three independent experiments that provided similar results.

**Figure 5:**
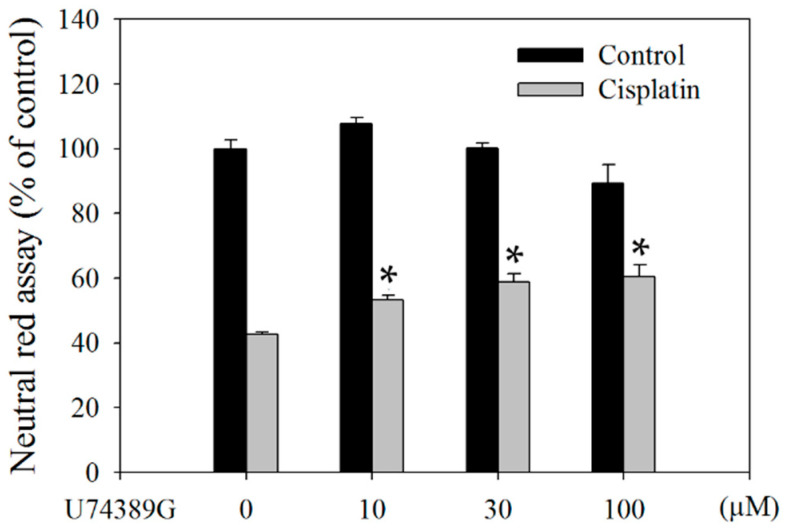
U74389G attenuated cisplatin-induced cell death in human SH-SY5Y cells. Cells were treated with or without 10 µM of cisplatin for 24 h and with or without U74389G in different doses for 1 h. Subsequently, cells were subjected to a neutral red assay. Viability is expressed as a percentage of controls. Data are presented as the mean ± standard deviation. Differences among multiple groups were evaluated using one-way analysis of variance. Differences between means were calculated using the Student-Newman-Keuls method and were considered significant at *p* < 0.05. * *p* < 0.05, compared with controls (*n* = 3–6).

Cisplatin significantly increased and reduced the fluorescence intensity of MitoSOX (Figure 4A) and tetramethylrhodamine, ethyl ester (TMRE) (Figure 4B), respectively. LJZT significantly attenuated these phenomena (Figure 4). Additionally, U74389G, a known antioxidant, also significantly attenuated cisplatin-induced cell death (Figure 5).

### 2.4. LJZT Attenuated Cisplatin-Induced Mitochondrial Pro-Apoptotic Factor Release

**Figure 6:**
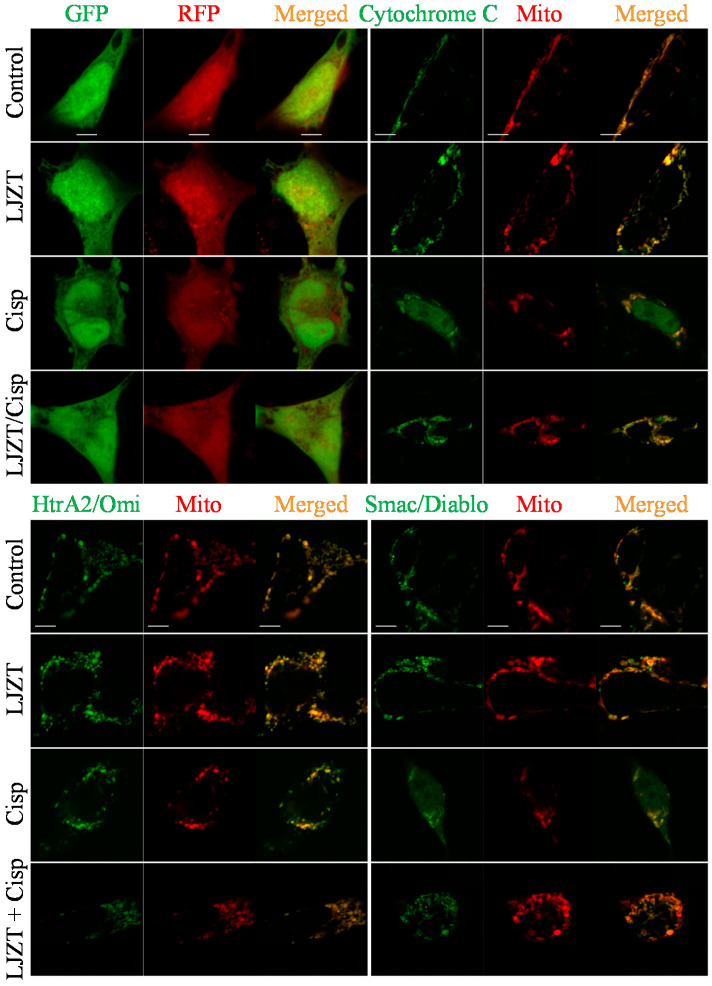
Effects of Liu Jun Zi Tang on cisplatin-induced mitochondrial pro-apoptotic factor release in human neuroblastoma (SH-SY5Y) cells. After co-transfection with pGFP/TagRFP (upper left panels), pGFP-cytochrome C/pDsRed-Mito (upper right panels), pHtrA2/Omi-GFP/pDsRed-Mito (lower left panels), or pSmac/Diablo-GFP/pDsRed-Mito (lower right panels) for 24 h, cells were pretreated with or without LJZT (100 µg/mL) for 1 h, then 10 µM of cisplatin (Cisp.) was administered for another 24 h. Cells were fixed and subjected to confocal analysis. In each treatment, 10–15 cells were randomly acquired. The bar represents 5 µm. These data represent one out of at least three independent experiments that provided similar results.

LJZT and cisplatin failed to influence the translocation of GFP and RFP (Figure 6, upper left panels). In our experiment, with or without LJZT treatment, mitochondrial pro-apoptotic factors, namely cytochrome C (Figure 6, upper right panels), HtrA2/Omi (Figure 6, lower left panels), and Smac/Diablo (Figure 6, lower right panels), were generally co-localized with mitochondria. However, cisplatin elicited the translocation of these factors (Figure 6), and LZJT pretreatment reduced these phenomena (Figure 6).

### 2.5. LJZT Reversed the Cisplatin-Induced Decreased Expression of PGC-1α

**Figure 7:**
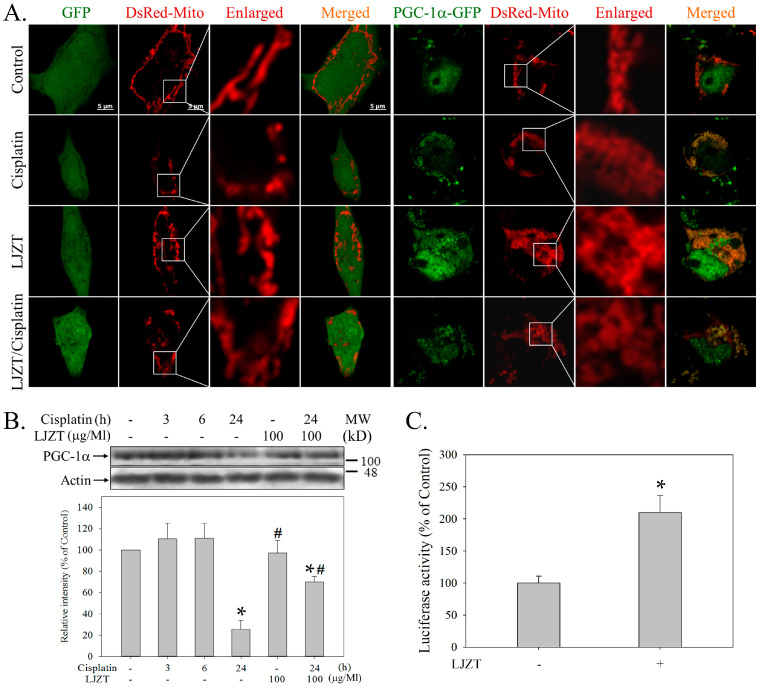
Effects of Liu Jun Zi Tang on peroxisome proliferator-activated receptor gamma coactivator 1 alpha (*PGC-1α*) expression during cisplatin intoxication in human neuroblastoma (SH-SY5Y) cells. (**A**) After transient transfection with pEGFP-N1 (expressed as a non-targeting green fluorescent protein)/pDsRed-Mito (expressed as a mitochondria-targeting red fluorescent protein) or pPGC-1α-GFP (expressed as a PGC-1α conjugating with green fluorescent protein)/pDsRed-Mito for 24 h, cells were pretreated with or without LJZT for 1 h, followed by the addition of cisplatin and incubation for another 24 h. Cells were then fixed, and images were acquired using a confocal microscope. At least three different fields were investigated for each treatment, and at least 10 cells per field were acquired. The bar represents 5 µm. (**B**) Cisplatin was administered at different intervals with or without LJZT pretreatment. Cells were then harvested and subjected to a Western blot analysis. The relative optical densities of the bands were quantified through densitometry relative to actin and normalized to the levels of the control condition. Data points represent the mean ± standard deviation. Differences among multiple groups were evaluated using one-way analysis of variance, and those between means were calculated using the Student-Newman-Keuls method with significance set at *p* < 0.05. * *p* < 0.05, compared with the control group. # *p* < 0.05, compared with the group treated with cisplatin for 24 h. (**C**) After the transient transfection of the *PGC-1α* promoter region (−2 Kb) was ligated with the luciferase gene for 24 h, cells were treated with or without LJZT for 4 h. Subsequently, cells were directly lysed, and their luciferase activities were measured. The Student’s *t*-test was used to compare differences between the two groups (*n* = 4/group). * *p* < 0.05, compared with the control group. These data represent one out of at least three independent experiments that provided similar results.

Cisplatin significantly induced mitochondrial fragmentation, which could be reversed by LJZT (Figure 7A, left panels). The overexpression of peroxisome proliferator-activated receptor gamma coactivator 1-alpha (*PGC-1α*) partly reversed cisplatin-induced mitochondrial fragmentation (Figure 7A, right panels). Cisplatin also reduced the expression of *PGC-1α*, which was also reversed by LJZT (Figure 7B). Additionally, LJZT significantly increased luciferase activity in the *PGC-1α* promoter region (Figure 7C).

### 2.6. Silencing of PGC-1α Gene Attenuated the Protection of LJZT

**Figure 8:**
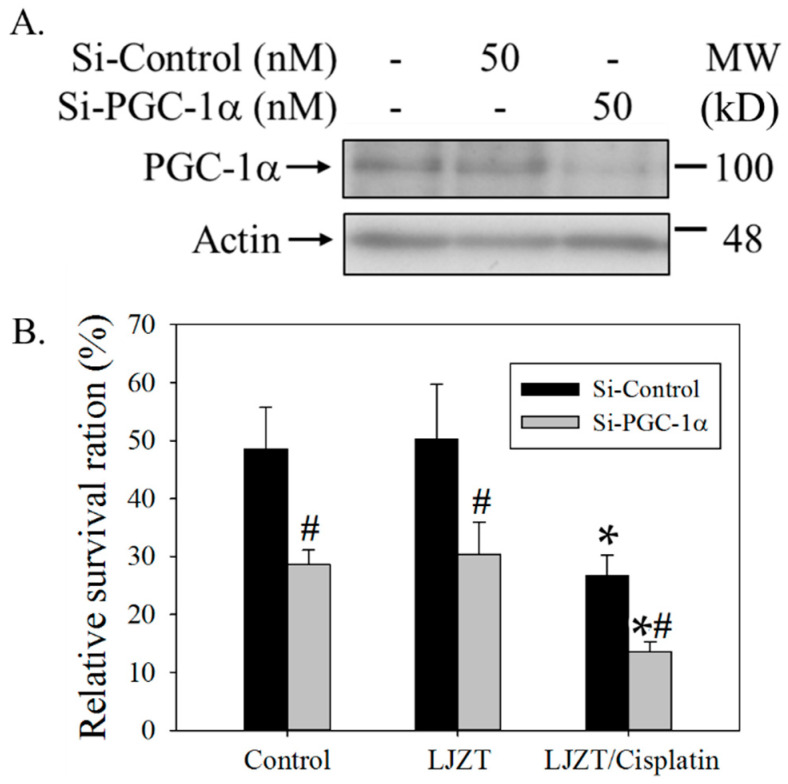
Effects of PGC-1α on the protection of Liu Jun Zi Tang in preventing cisplatin-induced cytotoxicity in human neuroblastoma (SH-SY5Y) cells. (**A**) Cells were harvested and subjected to a Western blot analysis with or without knockdown of the *PGC-1α* gene by siRNA (Si-*PGC-1α*) for 48 h. A control siRNA (Si-Control) was also used as a negative control. (**B**) Cells silenced by the control siRNA (Si-Control) or PGC-1α siRNA (Si-*PGC-1α*) were pretreated with LJZT for 1 h and then cisplatin for another 24 h. Subsequently, cells were subjected to a neutral red assay. Viability was calculated as a percentage with respect to its control group. In each gene-silenced group, the relative survival ratio was calculated from the viability of cells subtracted from that of the cells treated with cisplatin alone. Data are presented as the mean ± standard deviation. Differences among multiple groups were evaluated using two-way analysis of variance, and those between means were calculated using the Student-Newman-Keuls method with significance set at *p* < 0.05. * *p* < 0.05, compared with its control group respectively (*n* = 3–6). # *p* < 0.05, compared with its Si-Control-treated group (*n* = 3–6).

Silencing the *PGC-1α* gene reduced the expression of *PGC-1α* (Figure 8A). The survival ratio of LJZT in preventing cisplatin-induced cytotoxicity was relatively attenuated in *PGC-1α*-silenced cells (Figure 8B).

## 3. Discussion

Herbal treatments, such as supplements and total extracts, are commonly used worldwide [22,23]. Patients undergoing chemotherapy increasingly use medicinal herbs [24,25] or obtain physicians’ advice regarding their use. Regarding chemotherapy-induced neuropathy, goshajinkigan, which is composed of 10 herbal medicines, attenuated oxaliplatin-induced neuropathy in clinical trials [26,27], supporting the aforementioned viewpoint [3]. Although LJZT and its related decoctions have been intensively investigated as complementary therapy for chemotherapy-induced side effects [28,29,30], whether LJZT can prevent cisplatin-induced neurotoxicity remains unknown. In this study, we prospectively demonstrated that cisplatin-induced thermal hyperalgesia in mice [31] could be attenuated by LJZT (Figure 1). Furthermore, we observed that LJZT exerted a counter-protective effect in killing cancer cells (Figure S2), further supporting the alternative use of LJZT in cancer therapy. The protective mechanism is described in the following paragraphs.

### 3.1. LJZT Prevented Cisplatin-Induced Neurotoxicity

To study the neuroprotective mechanism of LJZT, a model of cisplatin-induced cell death was established in a neuron-like SH-SY5Y human cell line. Cisplatin was observed to induce cell death and the cleavage of PARP and caspase 3 (Figure 2), indicating an apoptotic form of cell death that was consistent with previous studies [32,33]. Furthermore, to the best of our knowledge, this is the first study to report that LJZT prevented cisplatin-induced neurotoxicity (Figure 1). This may partly explain the protective effects of LJZT-related decoctions in attenuating platinum compound-induced neuropathy, as described previously [3,4,5,6]. Although SH-SY5Y have been used in pain studies [32], which was supported by another study [34], the results required further investigation. To determine whether LJZT counteracts the therapeutic effects of cisplatin, human colon and lung cancer cells were analyzed, and it was found that LJZT failed to prevent the toxic effects of cisplatin, but induced cell death at higher doses (Figure S2), suggesting the efficacy of an appropriate decoction for complementary therapy.

### 3.2. LJZT Attenuated Cisplatin-Induced Cytosolic and Mitochondrial Free Radical Formation

Although interacting with other macromolecules, such as DNA, might be beneficial in treating cancers [35], cisplatin-induced free radical formation has been regarded as the toxic mechanism that induces side effects, such as nephrotoxicity, ototoxicity, and neurotoxicity [15,36]. We observed that cisplatin-induced cytosolic (Figure 3) and mitochondrial (Figure 4) free radical formation, and a known antioxidant (U74389G), which prevents oxidative stress-induced apoptosis [37], attenuated cisplatin-induced cytotoxicity (Figure 5). This partly explains the cisplatin-induced oxidative stress in SH-SY5Y cells [38] and LJZT-mediated attenuation of cisplatin-induced thermal hyperalgesia (Figure 1). Currently, mechanisms underlying cisplatin-induced free radical formation have not been conclusively delineated [39] or attributed to interactions with DNA [40], endoplasmic reticulum [41], NADPH oxidase [42], or mitochondria [43,44]. Furthermore, the mechanism through which LJZT attenuated cisplatin-induced cytosolic and mitochondrial free radical formation was not clear in this study. Accordingly, LJZT regulates cisplatin-induced side effects by potentiating ghrelin [45] and antagonizing the 5-HT_2B_ receptor [46]. Ghrelin [47] and 5-HT_2B_ receptor [48] signaling both mediate free radicals. Thus, determining whether LJZT can attenuate free radical formation through the regulation of ghrelin and 5-HT_2B_ receptor signaling in neuronal cells is an interesting research direction.

### 3.3. LJZT Attenuated Cisplatin-Induced Mitochondrial Dysfunction and PGC-1α Down-Regulation

Mitochondrial dysfunction, such as increased free radical formation [49] and pro-apoptotic factor release [37], plays a significant role in mediating apoptotic cell death. Thus, we revealed that except for mitochondrial free radical formation (Figure 4A) and pro-apoptotic factor release (Figure 6), cisplatin resulted in decreased mitochondrial membrane potential (Figure 4B) and mitochondrial fragmentation (Figure 7A), which was consistent with previous findings [50], and elicited mitochondrial dysfunction. This dysfunction has also been observed in the liver [51], dorsal root ganglion neurons [52], and nephrons [53]. Pathological changes in mitochondria, occurring due to cisplatin nephrotoxicity, are mainly triggered by mitochondrial DNA damage responses, pro-apoptotic protein attack, mitochondrial dynamics disruption, and oxidative stress [54]. Thus, the functional alteration of mitochondria during cisplatin intoxication tends to be a pivotal event for cell death [55,56].

In this study, we observed cisplatin-induced down-regulation of PGC-1α (Figure 7B), which is consistent with the finding of a previous study on renal cells [57]. Because PGC-1α is a master regulator of mitochondrial biogenesis, we analyzed the involvement of PGC-1α in this model. Notably, *PGC-1α* overexpression significantly attenuated cisplatin-induced mitochondrial fragmentation (Figure 7A, right panels). Because LJZT also attenuated cisplatin-induced mitochondrial fragmentation (Figure 7A) and PGC-1α downregulation (Figure 7B), we further examined the role of PGC-1α in the mediation of LJZT. As expected, LJZT activated the *PGC-1α* promoter (Figure 7C), thus revealing the mechanisms of LJZT in preventing cisplatin-induced mitochondrial fragmentation and PGC-1α downregulation. The silencing of the *PGC-1α* gene (Figure 8A) that resulted in attenuating the protection of LJZT (Figure 8B) may further strengthen the evidence that PGC-1α is a major signal transduction pathway for LJZT in antagonizing cisplatin-induced cytotoxicity. Thus, the mechanism through which LJZT attenuates free radicals could also be partly explained because PGC-1α buffers oxidative stress [58].

Mitochondrial disorders [20,21], such as impaired mitochondrial dynamics [59,60], have recently been proposed to play a role in mediating chemotherapy-induced neuropathy [61]. Thus, for the protection of LJZT in attenuating cisplatin-induced neuronal cell death (Figure 2), mitochondrial fragmentation (dynamics) (Figure 7), and hyperalgesia (Figure 1), LJZT might have therapeutic potential for cisplatin-induced neuropathy by regulating mitochondrial functioning. Therefore, the correction of mitochondrial functioning should be considered in the prevention and treatment of chemotherapy-induced neuropathy [62]. However, additional studies should be conducted to support this viewpoint.

Currently, neurotoxicity remains the most common dose-limiting toxicity of some chemotherapeutic compounds (such as cisplatin or its platinum compounds), hampering their clinical use [19,63]. However, effective treatments for preventing and treating this phenomenon are limited [64,65]. Because the toxic effects of cisplatin (or its platinum compounds) involve multiple organelles and mechanisms [36,66], a single-purpose or target-based drug is unlikely to treat these effects [6]. Thus, the use of traditional medicines with multiple ingredients, such as LJZT [67], as supporting supplements for attenuating chemotherapy-induced side effects, have drawn increasing attention [6,68,69]. Besides, although herbal Traditional Chinese Medicine (TCM) generally is regarded as natural and assumed to be safe, the side effects, such as hepatotoxicity, should also take into consideration during long term treatment. In LJZT, Bai Zhu, Fu Ling, Ban Xia, Ren Shen, or Sheng Jiang has also been prescribed in other formulae. However, these formulae, such as Chai Hu Tang, Long Dan Xie Gan, and Kamishoyosan, have been reported to cause hepatotoxicity [70,71,72]. Therefore, upon treatment cessation, LJZT should be used with strict regulatory surveillance. Finally, although our data supported the benefits of LJZT in treating cisplatin-induced neurotoxicity, additional detailed mechanisms are necessary to demonstrate its complemental use.

## 4. Materials and Methods

### 4.1. Reagents

All reagents were purchased from Sigma-Aldrich/Merk (St. Louis, MO, USA), except where specified otherwise. Anti-PARP (9542) and -caspase-3 (9662) were purchased from Cell Signaling (Beverly, MA, USA). Anti-actin (MAB1501) and anti-PGC-1α (ST1202) antibodies were purchased from Millipore (Billerica, MA, USA). The siGENOME SMART pool siRNAs and DharmaFECT transfection reagents were purchased from Thermo Scientific (Waltham, MA, USA). Dulbecco’s modified Eagle medium (DMEM)/F12 and fetal bovine serum (FBS) were purchased from GE Healthcare (Logan, UT, USA). PRO-PREP™ protein extraction solution was purchased from iNtRON Biotechnology (Seoul, Korea). Apart from pDsRed-Mito (expressed as a red fluorescent mitochondrial marker) and pEGFP-N1 (expressed as an enhanced green fluorescent protein), which were purchased from Clontech Laboratories (Mountain View, CA, USA), and pTagRFP-C (expressed as a red fluorescent protein), which was obtained from Evrogen (Moscow, Russia), all plasmids were purchased from Addgene (Cambridge, MA, USA). The luciferase reporter assay kit was purchased from BD Biosciences Clontech (Palo Alto, CA, USA).

### 4.2. LJZT Preparation

Fu Ling, Bai Zhu, Ban Xia, Gan Cao, Chen Pi, Sheng Jiang, and Da Zao were purchased from Yuanfang Pharmaceutical Co. (Taipei, Taiwan), and Ren Shen was acquired from Tianshun Herbal Pharmacopeia (Taipei, Taiwan). These herbs (Table 1) were authenticated by the I-Jung Lee Assistant Research Fellow and Herbarium Curator at the National Research Institute of Chinese Medicine, Ministry of Health and Welfare, Taiwan (NRICM). The voucher specimens were also verified according to the Taiwan Herbal Pharmacopeia (2013) and deposited in the herbarium of the NRICM. Each single herb and combined herbs of LJZT were immersed in distilled water (1:7, g/mL) for 30 min and refluxed for 40 min; then the aqueous extract was filtered and the residue was refluxed for 25 min with distilled water (1:5, g/mL) under the same conditions. These two extracts were successively combined, concentrated, and lyophilized to yield a dried powder, which was stored at 4 °C before use. The biochemical fingerprint of LJZT and its composition were determined using an ultra-performance liquid chromatography photodiode array (Figure S1).

### 4.3. Cell Culture

Human neuroblastoma SH-SY5Y cells were maintained in DME/F12 supplemented with 10% (*v*/*v*) FBS and incubated in a 5% CO_2_ incubator at 37 °C.

### 4.4. Survival Assays

A neutral red uptake assay [73] with a slight modification [74] was adopted to measure cell viability. In brief, after treatment, cells were loaded with 50 μg/mL neutral red and incubated at 37 °C for 2 h. Cells were then washed once with 200 μL of phenol red-free medium and added to 100 μL of destaining solution (50% ethanol [96%], 49% deionized H_2_O, and 1% glacial acetic acid). The absorbance at 540 nm of each well was measured using a micro-enzyme-linked immunosorbent assay reader.

### 4.5. Cisplatin-Induced Neuropathy and Tail Flick Assay

Cisplatin-induced neuropathy of a mouse model was modified from a previously described method [31] and approved by the Institutional Animal Care and Use Committee of the NRICM (103-809-1). Briefly, 8-week-old Balb/c mice, obtained from the National Laboratory Animal Center, Taiwan, were randomly housed 6 to a cage and habituated for 2 weeks to prevent stress-induced analgesia. Cisplatin (Sigma, St. Louis, MO, USA) was dissolved in normal saline and prepared each day. Mice received either cisplatin (2.3 mg/kg) or a vehicle (normal saline) intraperitoneally for 5 days, followed by a 9-day rest, and administration was repeated for another 5 days. LJZT was administered orally once a day, 5 days per week, for 3 cycles. The dosages of LJZT were equal to the dosage used in adult humans, based on the translation equation [75]. A tail-flick assay was used to monitor cisplatin-induced neuropathy as described previously [76]. The latency of the tail withdrawal was measured by a tail-flick test analgesia meter purchased from IITC Life Science Inc. (Victory Blvd Woodland Hills, CA, USA).

### 4.6. Western Blot Analysis

A Western blot analysis was performed as described previously [77]. In brief, cells were rinsed with ice-cold phosphate buffered saline (PBS) and lysed in an ice-cold lysis buffer (20 mM 4-(2-hydroxyethyl)-1-piperazineethanesulfonic acid, 1 mM dithiothreitol, 20 mM EGTA, 10% glycerol, 50 mM β-glycerophosphate, 10 mM NaF, 1% Triton X-100, 1 mM PMSF, 1 mM Na_3_VO_4_, 2 μM aprotinin, 100 μM leupeptin, 2 μM pepstatin, and 0.5 μM okadaic acid). After sonication, cell debris was removed through centrifugation at 14,000 rpm for 10 min, and the supernatant was used for the Western blot analysis. Equal amounts of the sample were separated by polyacrylamide gel electrophoresis. Resolved proteins (25 μg/lane) were then electroblotted onto Immobilon PVDF membranes (Millipore, Bedford, MA, USA). Membranes were blocked with 5% skim milk or 3% bovine serum albumin, then sequentially incubated with the first and second antibodies overnight at 4 °C for 1 h at room temperature, respectively. After washing, blots were processed for visualization using an enhanced chemiluminescence system (Pierce). Blots were then exposed to a Fuji medical X-ray film (Super RX-N, FUJIFILM Corporation, Tokyo, Japan) to obtain fluorographic images.

### 4.7. Measurement of Intracellular Reactive Oxygen Species

Intracellular peroxide and superoxide formations were measured through staining with 5-(and 6-)carboxy-2’,7’-dichlorodihydrofluorescein diacetate (CO_3_-H_2_DCFDA) and DHE, respectively. DHE, due to its ability to freely permeate cell membranes, is used extensively to monitor superoxide production. When reacting with superoxide anions, it forms a red fluorescent product, HE. CO_3_-H_2_DCFDA is a non-fluorescent and cell-permeable analog that is converted into CO_3_-H_2_DCF fluorescein after intracellular deacetylation, and oxidized into highly fluorescent CO_3_-DCF. In brief, after 10 μM DHE or 25 μM CO_3_-H_2_DCFDA staining for 15 min, images of the cells were taken with the same gain and fixed exposure times using a Zeiss Axiovert 200 M inverted fluorescence microscope (Göttingen, Germany). At least three fields were acquired and calculated for each treatment and at least 15–30 cells per field were used for quantifications. The fluorescent intensities of the pictures were quantitated using ImageJ (Lakes, NJ, USA) software with the background subtracted.

### 4.8. Measurement of Mitochondrial Reactive Oxygen Species (ROS) and Membrane Potential

Cells were washed with Hank’s Buffered Salt Solution and stained with 5 μM of Mito-SOX (a marker of mitochondrial superoxide) for 10 min or 100 nM of TMRE (a marker of mitochondrial membrane potential) for 15 min, where fluorescence intensity represents mitochondrial ROS and membrane potential. Cells were then employed for image acquisition with the same gain and fixed exposure times with a Zeiss Axiovert 200 M inverted fluorescence microscope. At least three fields were acquired and calculated for each treatment, and 15–30 cells per field were used for quantifications. The fluorescence intensities of the pictures were quantified using ImageJ with the background subtracted.

### 4.9. Transient PGC-1α Gene Silencing

The non-targeting (NT) control siRNA and SMART pools of siRNAs that target human *PGC-1α* were used to silence the expression of *PGC-1α*, according to standard protocols [37].

### 4.10. Transient Transfection, Luminescence, and Image Detections

The jetPRIME^®^ transfection reagent kit (Polyplus-transfection SA, Illkirch, France) was used to transfer plasmids into cells, as described in the experimental protocol. Generally, 0.5 μg DNA combined with 50 μL of buffer and 1 μL of reagent were applied to each well in the 24-well plates (1.5 × 10^5^ cells/well). After transfection for 24 h, cells were treated with reagents for different intervals and then directly lysed with PRO-PREP™ protein extraction solution (100 µL/well) on ice for 10 min. Cell lysates (100 µL) and luciferase substrates (50 µL) were added, and luminescence was detected using a SpectraMax M5 microplate reader (Molecular Devices, Sunnyvale, CA, USA). Alternatively, other cells (6 × 10^4^ cells/well) grown on sterile glass coverslips were also transfected with DNA, using the same protocol. However, after treatment, cells were fixed for 10 min with 4% paraformaldehyde at room temperature, then thrice rinsed with PBS. Coverslips were further mounted on microscope slides with Aqua Poly-Mount (Polysciences, Warrington, PA, USA). Coverslip images were observed with a confocal microscope (LSM 780, Carl Zeiss, Göttingen, Germany).

### 4.11. Statistical Analysis

The significance of the drug treatments was determined by a Student’s *t*-test or one- or two-way analysis of variance. *Post hoc* comparisons between means were conducted using the Student-Newman-Keuls procedure. Statistical significance was set at *p* < 0.05.

## 5. Conclusions

We revealed that LJZT can be a potential adjunct therapy, and LJZT-mediated anti-oxidation and mitochondrial functioning (such as membrane potential and biogenesis) may be protective mechanisms for alleviating cisplatin-induced neurotoxicity.

## Figures and Tables

**Table 1:** Contents of Liu Jun Zi Tang (LJZT).

Chinese Name	Botanical Name	Common Name	Weight (g)	Part Used or Processed
Bai Zhu	*Atractylodes mocrocephala* Kioidz.	Atractylodes Mocrocephala Rhizoma	5.0	Rhizome
Ban Xia	*Pinellia ternate* (Thunb.) Breit.	Pinelliae Rhizoma	5.0	Rhizome Gingered
Chen Pi	*Citrus reticulate* Blanco	Tangerine Peel	2.5	Peel
Da Zao	*Ziziphus jujube* Mill.	Jujubae Fructus	2.5	Fruit
Fu Ling	*Poria cocos* (Schw.) Wolf	Poria	5.0	Sclerotium
Gan Cao	*Glycyrrhiza uralensis* Fisch	Glycyrrhizae Radix et Rhizoma	2.5	Root and Rhizome
Ren Shen	*Panax jinseng* C.A. Mey	Ginseng Radix et Rhizoma	5.0	Root and Rhizome
Sheng Jiang	*Zingiber officinale* Rosc.	Zingiberis Rhizoma Recens	2.5	Rhizome

## Supplementary Materials

Supplementary materials can be found at https://www.mdpi.com/1422-0067/19/4/1258/s1.
