# Correction: Chiou et al. Liu Jun Zi Tang—A Potential, Multi-Herbal Complementary Therapy for Chemotherapy-Induced Neurotoxicity. *Int. J. Mol. Sci.* 2018, *19*, 1258

**DOI:** 10.3390/ijms27146097

**Published:** 2026-07-08

**Authors:** Chun-Tang Chiou, Kaw-Chen Wang, Ying-Chen Yang, Chuen-Lin Huang, Sien-Hung Yang, Yao-Haur Kuo, Nai-Kuei Huang

**Affiliations:** 1 National Research Institute of Chinese Medicine, Ministry of Health and Welfare, Taipei 11031, Taiwan; ctchiou@nricm.edu.tw (C.-T.C.); kuoyh@nricm.edu.tw (Y.-H.K.); 2 Department of Neurology, Cardinal-Tien Hospital, New Taipei City 231, Taiwan; aronkcwang@gmail.com; 3 Department of Biotechnology and Animal Science, National Ilan University, Ilan 260, Taiwan; ycyang@niu.edu.tw; 4 Medical Research Center, Cardinal Tien Hospital, Hsintien, New Taipei City 231, Taiwan; chuenlinhuang@hotmail.com; 5 Graduate Institute of Physiology & Department of Physiology and Biophysics, National Defense Medical Center, Taipei 11031, Taiwan; 6 School of Traditional Chinese Medicine, Chang Gung University, Taoyuan City 333, Taiwan; dryang@adm.cgmh.org.tw; 7 Graduate Institute of Integrated Medicine, College of Chinese Medicine, China Medical University, Taichung 404, Taiwan; 8 Ph.D. Program for Neural Regenerative Medicine, College of Medical Science and Technology, Taipei Medical University, Taipei 11031, Taiwan

**Error in Figure**

In the original publication [1], there was a mistake in Figure 7B as published. Recently, we noticed a portion of a band appears to have been used in two different Figures. This error was unintentional and occurred during figure assembly. The corrected version of Figure 7B appears below.

**Text Correction**

There was an error in the original publication. In Section 4.11, Statistical Analysis, the notation “*p* < 0.5” should be revised to “*p* < 0.05”.

A correction has been made to 4. Materials and Methods, 4.11. Statistical Analysis, Paragraph 1:

The significance of the drug treatments was determined by a Student’s *t*-test or one- or two-way analysis of variance. *Post hoc* comparisons between means were conducted using the Student-Newman-Keuls procedure. Statistical significance was set at *p* < 0.05.

The authors state that the scientific conclusions are unaffected. This correction was approved by the Academic Editor. The original publication has also been updated.

## Figures and Tables

**Figure 7:**
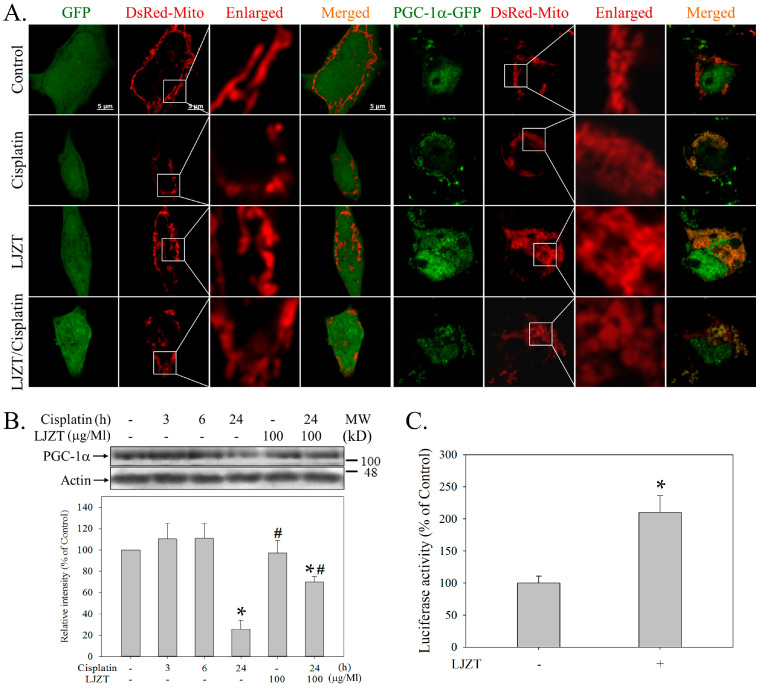
Effects of Liu Jun Zi Tang on peroxisome proliferator-activated receptor gamma coactivator 1 alpha (*PGC-1α*) expression during cisplatin intoxication in human neuroblastoma (SH-SY5Y) cells. (**A**) After transient transfection with pEGFP-N1 (expressed as a non-targeting green fluorescent protein)/pDsRed-Mito (expressed as a mitochondria-targeting red fluorescent protein) or pPGC-1α-GFP (expressed as a PGC-1α conjugating with green fluorescent protein)/pDsRed-Mito for 24 h, cells were pretreated with or without LJZT for 1 h, followed by the addition of cisplatin and incubation for another 24 h. Cells were then fixed, and images were acquired using a confocal microscope. At least three different fields were investigated for each treatment, and at least 10 cells per field were acquired. The bar represents 5 µm. (**B**) Cisplatin was administered at different intervals with or without LJZT pretreatment. Cells were then harvested and subjected to a Western blot analysis. The relative optical densities of the bands were quantified through densitometry relative to actin and normalized to the levels of the control condition. Data points represent the mean ± standard deviation. Differences among multiple groups were evaluated using one-way analysis of variance, and those between means were calculated using the Student-Newman-Keuls method with significance set at *p* < 0.05. * *p* < 0.05, compared with the control group. # *p* < 0.05, compared with the group treated with cisplatin for 24 h. (**C**) After the transient transfection of the *PGC-1α* promoter region (−2 Kb) was ligated with the luciferase gene for 24 h, cells were treated with or without LJZT for 4 h. Subsequently, cells were directly lysed, and their luciferase activities were measured. The Student’s *t*-test was used to compare differences between the two groups (*n* = 4/group). * *p* < 0.05, compared with the control group. These data represent one out of at least three independent experiments that provided similar results.
