# Supplementary material for: Liu Jun Zi Tang—A Potential, Multi-Herbal Complementary Therapy for Chemotherapy-Induced Neurotoxicity
Source: Int J Mol Sci. 2018 Apr 23;19(4):1258. doi: 10.3390/ijms19041258 (PMC5979528; doi:10.3390/ijms19041258)
Supplement: Supplementary file 1 [file ijms-19-01258-s001.pdf]

Supplementary Materials:

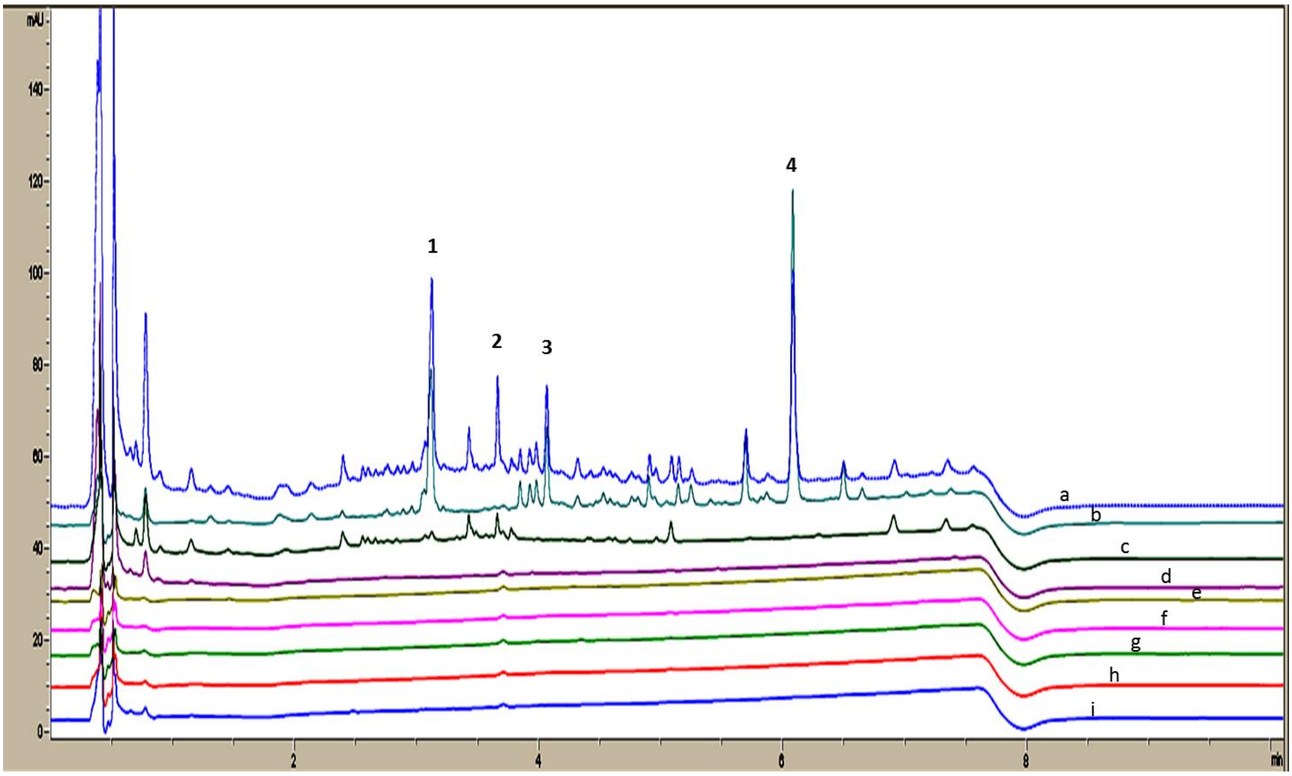

Figure S1. The fingerprint of LJZT and its compositions. All lyophilized hot water extracts of LJZ decoction and each herb were weighed about 0.5 g and dissolved in 50 mL 50% MeOH. The samples were soaked for 2 hours at room temperature, ultrasonic extracted for 60 min (300 W, 40 kHz), and then filtrated through a 0.22 μm membrane before loading on UPLC. UPLC was performed using an Agilent 1200 Infinity Series (Santa Clara, CA, USA) equipped with Zorbax RRHP Eclipse Plus C18 column (2.1x50 mm, 1.8 μm). The mobile phase included A (0.1% formic acid in H_2O) and B (0.1% formic acid in acetonitrile). The gradient elution program was B channel 10-10% (0-1 min), 10-30% (1-3.5 min), 30-50% (3.5-7 min), 50-10% (7-7.1 min), and 10-10% (7-10 min). Flow rate was 0.3 mL/min. The UV detector wavelength was 254 nm. Profile a, b, c, d, e, f, g, h, and i respectively represented LJZT, Gan Cao, Chen Pi, Bai Zhu, Sheng Jiang, Fu Ling, Ban Xia, Ren Shen, and Da Zao. Peak 1, 2, 3, and 4 respectively indicated liquiritin, naringin, hesperidin, and glycyrrhizic acid. According to the Pharmacopoeia of the People's Republic of China 2010 (Volume I, page 898), hesperidin was use a requiring reference index of Xiang-Sha-Liujun Wan which contains two more herbs (Rhizome of *Rosa banksiae* R. Br. and fruit of *Amomum villosum* Lour.) than LJZT has.

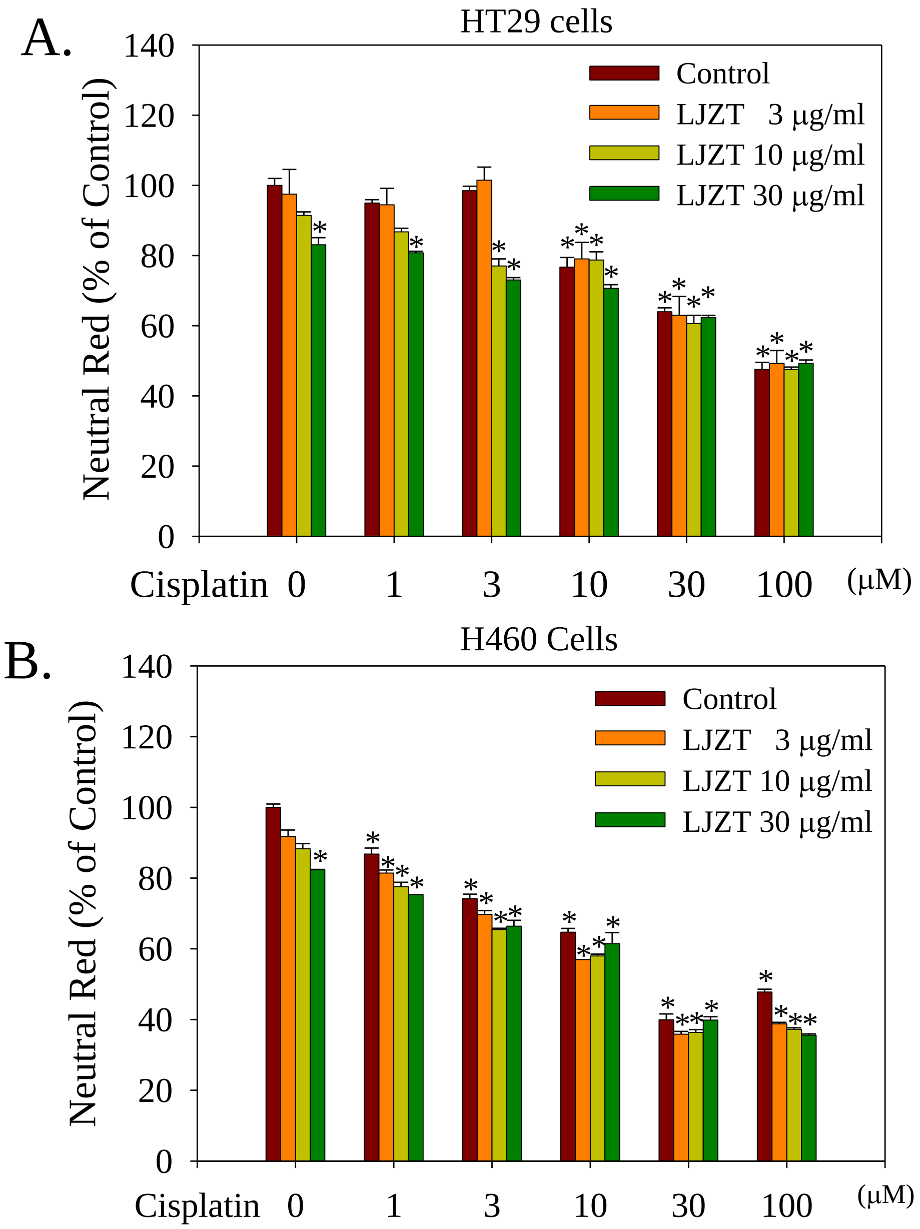

Figure S2. **LJZT failed to prevent cisplatin-induced toxicity in two cancer cell lines.** (A) human colon adenocarcinoma grade II (HT29) cells and (B) human non-small cell lung carcinoma cells (H460) with or without treating with LJZT in different dosages for 1 h and with or without treatment with cisplatin for another 24 h. Cells were then subjected to a neutral red assay. Viability is expressed as a percentage of controls. Data is presented as the mean ± SD. *$p < 0.05$, compared to controls (n = 3~6).
